# Crop rotation-driven changes in secondary metabolites of potato rhizosphere soil exert stronger regulation on soil microbial community

**DOI:** 10.3389/fmicb.2026.1768797

**Published:** 2026-03-05

**Authors:** Jinjin Li, Qingcheng Li, Mantang Wang, Shuqing Xu, Danju Zhang

**Affiliations:** 1 College of Tourism, Resources and Environment, Zaozhuang University, Zaozhuang, China; 2 Triticeae Research Institute, Sichuan Agricultural University, Chengdu, Sichuan, China; 3 College of Forestry, Sichuan Agricultural University, Chengdu, Sichuan, China

**Keywords:** crop rotation, potato planting, rhizosphere, secondary metabolites, soil microbial community

## Abstract

**Introduction:**

Crop rotation promotes ecological effects and production by regulating belowground processes, particularly the shaping of the rhizosphere soil microbiome. Rhizosphere metabolites are a key driver of belowground processes and play a crucial role in shaping soil microbial community composition. However, the rhizosphere metabolites of different potato rotations have rarely been reported, and the regulation of key metabolites on the rhizosphere soil microbiome remains unclear.

**Methods:**

This study measured agronomic traits of potatoes, collected potato rhizosphere soils from three crop rotations, including potato monoculture (P-P), maize (*Zea mays*)-potato rotation (M-P), cowpea (*Vigna unguiculata*)-potato rotation (V-P), to determine rhizosphere soil metabolites and analyze defense metabolites, and assess the soil bacterial and fungal diversity and community composition.

**Results:**

Compared to monoculture, the potato rotations had positive effects on growth and yield. Potato rotations had more primary metabolites, such as amino acids and carbohydrates and conjugates, but significantly reduced secondary metabolites with defensive functions in rhizosphere soils including phenols and other benzene derivatives, flavonoids, alkaloids and other N-containing compounds, and terpenoids. Potato rotation systems supported higher diversity of bacteria and fungi and enriched beneficial bacteria such as biocontrol, nitrogen fixation, C degradation, denitrification, and pollutant degradation bacteria, while suppressing pathogenic fungi in the rhizosphere soils. Rhizosphere soil metabolites strongly correlated with the microbial community composition. The secondary metabolites, which are predominantly alkaloids, terpenoids, and flavonoids, exerted a dominant regulatory effect on the composition of soil microbial community.

**Discussion:**

These results demonstrate the important regulation of rhizosphere metabolites on soil microbial community composition, deepening our understanding of the benefits of crop rotation via the belowground effect.

## 1 Introduction

Crop rotation, as a sustainable tillage mode, is becoming popular agricultural planting practice (Fang et al., 2021; Wang Y. et al., 2023; Wang Y. Z. et al., 2023). In reasonable rotation systems, the legacy effect of previous crops has positive impacts on the growth and production of subsequent crops (Somenahally et al., 2018; Zhao et al., 2020; Wang et al., 2025). Abundant evidence has indicated that crop rotations can break life cycles of pathogen and pest, improve soil nutrient availability, and reduce the need for agrochemical resources, thereby promoting the overall quality and productivity (Chen et al., 2001; Leandro et al., 2018; Zhang et al., 2023; Liu M. et al., 2025; Liu X. X. et al., 2025; Liu Y. L. et al., 2025). The positive effects of rotation cropping systems are increasingly being demonstrated to be mainly driven by the belowground interaction processes of pants-soil-microbes (Benitez et al., 2021; Wang Y. et al., 2023; Wang Y. Z. et al., 2023; Hu et al., 2025; Zhang et al., 2025). However, the underlying belowground mechanisms of these processes remain unclear, thereby limiting the understanding of the yield benefits of crop rotation.

Soil microorganisms can mediate essential belowground ecological processes in cropping systems through various mechanisms, including accelerating soil nutrient cycling, increasing soil nutrient availability for plants, synthesizing plant growth promoting compounds, eliciting plant defense responses, and directly suppressing soil pathogens via competition or antibiosis (Jacoby et al., 2017; Kwak et al., 2018; Stringlis et al., 2018; Benitez et al., 2021; Wang Y. et al., 2023; Wang Y. Z. et al., 2023). Therefore, the diversity and community composition of soil- and plant-associated microbes are tightly associated with plant performance (Pretty et al., 2018; Benitez et al., 2021). Numerous studies have demonstrated that the abundance, diversity, and composition of soil microbes alter in crop rotation compared to continuous cropping (Zhou et al., 2022; Ding et al., 2024; Liu et al., 2024). For example, a meta-analysis of 122 studies on crops such as soybean, sorghum, corn, and wheat revealed that rotational diversity can increase soil microbial biomass by more than 20%, which is primarily attributed to enhanced soil nutrient cycling (McDaniel et al., 2014). In soybean-maize rotation, beneficial bacteria and fungi were enriched and the abundance of pathogens was suppressed relative to continuous maize cropping (Benitez et al., 2021). These changes in soil microorganisms well explain the increased plant biomass and yield observed in rotation ecosystems (Venter et al., 2016; Jiang et al., 2024; Liu M. et al., 2025; Liu X. X. et al., 2025; Liu Y. L. et al., 2025). While the importance of soil microorganisms in mediating the effects of rotation on crop productivity has been widely demonstrated, the underlying other crucial belowground processes regulating these effects remain poorly understood. For instance, the contribution of rhizosphere metabolites and their interactions with the microbiome to crop production in crop rotation systems is uncertain.

Rhizosphere soil metabolites, which mainly derive from plant root exudates, root litter, and microbial metabolism, are also key drivers of belowground processes, tightly connecting the pant-soil-microbe interactions (Haichar et al., 2014; Hu et al., 2018; Fan et al., 2022; Ma et al., 2022; Wang Y. et al., 2023; Wang Y. Z. et al., 2023). The rhizosphere metabolic pool comprises a variety of chemical classes, such as sugars, organic acids, and secondary metabolites (Bais et al., 2006; Hu et al., 2018; Kong et al., 2019). Defensive secondary metabolite compounds in rhizosphere soil such as phenolic compounds, terpenoids, and alkaloids, are mainly produced by plants or microorganisms under biotic or abiotic stress to mitigate adverse environments. These compounds can act as allelochemicals exerting directly inhibitory effects on plants (Kong et al., 2018, 2019). For instance, they can influence crop growth by interfering with root development and hormone synthesis (Kato-Noguchi and Ino, 2003; Liu et al., 2016). Some metabolites can also regulate soil environmental conditions and the microbial community structure such as pathogen and mutualist, thereby affecting crop performance in different ways (Kovacs, 2023; Zhou et al., 2023; Han et al., 2024; Jiang et al., 2024). The diversity and composition of rhizosphere soil metabolites vary significantly between different crop rotation systems (Lu et al., 2019; Liu M. et al., 2025; Liu X. X. et al., 2025; Liu Y. L. et al., 2025). Previous crop legacy effects such as the compounds remaining in the soil, soil microorganisms and food webs, and their triggered metabolite secretions of current crops, all cause the variation in rhizosphere soil metabolites. Therefore, as crop community changes among different rotation regimes, variations in the rhizosphere metabolite composition occur (Haichar et al., 2008; Hu et al., 2018; Lu et al., 2019).

Rhizosphere soil metabolites can directly influence the diversity and composition of soil microbial community (Hu et al., 2018; Ding et al., 2021; Bi et al., 2022; Wang Y. et al., 2023; Wang Y. Z. et al., 2023; Jiang et al., 2024). Sucrose, glycerol-3-galactoside, N-carbanoylaspartate, and vitamins provide a readily available source of carbon and nutrients for the growth and reproduction of rhizosphere microorganisms (Badri and Vivanco, 2009; Ding et al., 2021). The metabolites such as alkaloid and 6-methoxybenzoxazolin as defensive secondary compounds inhibit the diversity and community structure of microbes (Martyniuk et al., 2006; Soltoft et al., 2007; Huang et al., 2022). For instance, the benzoxazinoids released by the roots of wheat and maize exert strong allelopathic effects on the activity and community composition of rhizosphere bacteria and fungi (Hu et al., 2018). Rhizosphere metabolites can also behave as chemical signals, either blocking or recruiting specific microbial taxa to the rhizosphere, triggering different effects on the plant performance (Akiyama and Hayashi, 2006; Kong et al., 2018; Murata et al., 2019). Flavonoids and their derivatives released by legumes as key signaling can recruit both mutualist and pathogen (Peters et al., 1986). Rhizosphere metabolites can influence soil microbes indirectly by modifying soil environmental factors (Haichar et al., 2014; Ding et al., 2021). Organic acids such as citric acid, malic acid, and malonic acid have a significant influence on soil pH (Hu et al., 2018; Ma et al., 2022). Sugars and amino acids serve as key inputs that increase labile soil organic matter (Zhuang et al., 2024). These changes reshape the soil habitat and food webs and ultimately affect rhizosphere microorganisms (Haichar et al., 2014; Li et al., 2023). Therefore, the interactions between rhizosphere metabolites and microbes play an important role in regulating belowground processes of cropping systems (Shen et al., 2020; Wang Y. et al., 2023; Wang Y. Z. et al., 2023). Exploring the dynamics and functions of rhizosphere soil metabolites, as well as the changes in soil microbial diversity and composition they mediate, will deepen our understanding of rotation-driven advantages of cropping systems.

Potato (*Solanum tuberosum*), globally ranked as the fourth food crop, provides a stable diet for people (Qin et al., 2022; Chen et al., 2023). However, successive potato cropping has been shown to bring a number of environmental threats, such as soil-borne diseases, loss of soil fertility, and reduced productivity (Qin et al., 2022; Ma et al., 2023). Crop rotation could potentially attenuate these adverse effects by improving soil physicochemical properties and enzyme activities, and mediating soil microbial community composition (Larkin et al., 2021; Wang Y. et al., 2023; Wang Y. Z. et al., 2023; Liu et al., 2024). For example, a crop rotation involving legumes and potatoes promotes soil fertility and healthy ecosystem, and modulates rhizosphere metabolites relative to continuous potato cropping (Wang Y. et al., 2023; Wang Y. Z. et al., 2023). Faba bean-potato-oat rotation improves soil microbial community structure and reduces potato black scurf incidence (Qin et al., 2022). Although some studies have documented the changes in microbial diversity and composition in potato rotation systems, the mechanisms by which changes in rhizosphere metabolites regulate microbiome communities in different rotation systems remain unclear. This research would provide an important explanation for advantages of potato rotation production.

In this study, three common potato rotation patterns in the study region including potato monoculture, maize (*Zea mays*)-potato, and cowpea (*Vigna unguiculata*)-potato were selected. Potato rhizosphere soil samples were collected from field experimental plots during the peak tuber bulking stage. We analyzed the variation in agronomic traits, rhizosphere soil metabolites, and soil microbiomes, revealed the critical differential metabolites and microbial taxa, as well as their interactions between different potato rotation systems. The objective of the study was to examine the regulation of changes in rhizosphere soil metabolites on soil microbial communities in different potato rotation systems. We tested the hypotheses that (1) potato rhizosphere soil metabolites profiles varied between rotation systems; (2) changes in rhizosphere metabolites profiles may significantly influence the soil microbial communities, with critical secondary metabolites exerting a predominant regulation on their diversity and composition.

## 2 Materials and methods

### 2.1 Experimental station

Tengzhou City, known as the “hometown of Potatoes” in China (116°49′-117°24′E, 34°50′-35°17′N), is located in the Shandong province. The study site has a temperate continental monsoon climate, with a mean annual precipitation of 773.1 mm and an average temperature of 13.6 °C. The soil is classified as fluvo-aquic soil, and its basic physicochemical characteristics were described in Table 1.

**Table 1:** Physiochemical characteristics of potato rhizosphere soil under different rotation systems.

Site	Soil pH	Soil organic matter (g/kg)	Soil total N (g/kg)	Soil total P (g/kg)	Soil available P (mg/kg)
P-P	6.00 ± 0.06c	7.95 ± 0.07b	0.81 ± 0.00b	1.90 ± 0.26a	20.36 ± 0.76a
M-P	6.54 ± 0.01b	8.20 ± 0.08b	1.25 ± 0.03a	0.75 ± 0.01b	18.10 ± 0.18b
V-P	6.74 ± 0.01a	15.85 ± 0.54a	1.07 ± 0.11ab	1.68 ± 0.01a	10.23 ± 0.18c

Different lowercase letters indicate significant differences between rotation patterns at *p* < 0.05; P-P, potato-potato rotation; M-P, maize-potato rotation; V-P, cowpea-potato rotation.

The study region typically cultivates potatoes in both spring and autumn each year. This system provides a summer growing window. The conditions during this period are favorable for both maize and cowpea. The maize-potato and cowpea-potato patterns have become the typical cropping rotation patterns in the study region. The following three treatments were used in the field experiment: potato-potato (P-P), maize-potato (M-P), and cowpea-potato (V-P). In P-P systems, the double potato planting is planted separately in spring and autumn. After planting spring potatoes, the maize and cowpea were planted before planting autumn potatoes in M-P and V-P systems, respectively. Then, each of the rotation patterns were managed under a fallow winter. The seed tubers of potato variety “Helan 15” used in the study were obtained from Shandong Grad Group Co., Ltd., China. The maize variety “Zhengdan958” and the cowpea variety “Zhijiang 28-2” were purchased from Shandong Denghai Seeds Co., Ltd., China. In the study region, these potato rotation patterns have lasted for 5 years. The crop rotation treatments were established, with each one conducted in the three plots with an area of 5.4 m × 12 m. According to the typical nutrient requirements of each crop, fertilizer was applied and the amounts were shown in Supplementary Table S1. Potatoes were planted in two rows per monopoly with the planting density of 7.5 × 10^4^ plants ha^−1^. Routine management was applied for weed and insect control throughout the whole growing period.

### 2.2 Rhizosphere soil sample collection

Rhizosphere soil samples were sampled in October 2024, during the peak tuber bulking stage of the potato crop. In each plot, five plants were randomly chosen and their complete roots still attached to plant were carefully excavated. Then, we separated rhizosphere soil that tightly adhered to the roots by shaking it off and transported it to the laboratory. Soil sample from same plot were homogenized into one composite sample. There were three biological replicates of soil samples per potato cropping pattern for subsequent analysis. The soil sample was then divided into two aliquots. One subsample of each rhizosphere soil sample was first frozen in liquid nitrogen and stored at −80 °C for ultra-performance liquid chromatography–tandem mass spectrometry (UPLC–MS/MS) analysis and high-throughput sequencing of the soil microbiome. The other soil sample was air-dried for physicochemical property analysis.

### 2.3 Measurement of agronomic traits and soil physicochemical properties

During the mature stage, five potato plants were randomly selected from each plot for determine the agronomic traits and yield. The height of the potatoes was measured from the soil surface to the apical meristem of the main stem using a measuring tape. The stem diameter was determined at the base of the main stem using a digital caliper. Total tuber number per potato was recorded at harvest. Total tuber yield was determined by harvesting all the plants in each plot (Susanna and Anna, 2018).

The soil pH was determined using a pH meter (Model PHS-2, INESA Instruments, Shanghai, China). Soil organic matter was determined by the potassium dichromate capacity. Soil total nitrogen was evaluated by the Kjeldahl method. Soil total phosphorus (P) was extracted using HCL, available P was extracted using Bray-1, and then the P concentration was determined using molybdenum antimony colorimetric method (Gu and Aiverson, 2020).

### 2.4 Rhizosphere soil metabolite extraction and data analysis

The metabolites in the rhizosphere soil samples were extracted using methanol. The samples were then vortexed and centrifuged to obtain a supernatant, which was used for liquid chromatography-mass spectrometry analysis (LC–MS). The LC analysis was performed using a Vanquish UHPLC system (Thermo Fisher Scientific, United States) equipped with an AXQUITY UPLC® HSS T3 (100 × 2.1 mm, 1.8 μm; Waters, Milford, MA, United States). Mass spectrometric detection of metabolites was performed using an Orbitrap Exploris 120 (Thermo Fisher Scientific, United States) with an ESI ion source. HCD scans with a normalized collision energy of 30% were used for MS/MS analysis. Redundant information in the MS/MS spectra was removed by dynamic exclusion (Zhang D. J. et al., 2022; Zhang J. X. et al., 2022).

The raw data were converted to mzXML format using Proteowizard software (Version 3.0.8789), and identification, filtration and alignment of the peaks were then processed using the XCMS package in R (Version 3.12.0) to obtain quantitative list of metabolites. The area normalization method was used for correction of the data to eliminate systematic errors. The metabolites were then identified by matching the following databases: HMDB,^1^ massbank,^2^ KEGG,^3^ LipidMaps,^4^ mzcloud^5^ and the metabolite database built by Panomix Biomedical Tech Co., Ltd. (Suzhou, China) (Kanehisa and Goto, 2000). Differential metabolites between the groups were screened using the OPLS-DA model with VIP values ≥ 1 and *P* values ≤ 0.05.

### 2.5 High-throughput sequencing and sequence processing

Total DNA of rhizosphere soil was extracted using the E. Z. N. A. ®Soil DNA Kit (Omega Bio-tek, Norcross, United States) following the manufacturer’s protocols. DNA concentrations were determined using a Nanodrop 2000 UV–Vis spectrophotometer (Thermo Scientific, Wilmington, United States) and quality was checked using 1% agarose gel electrophoresis. The primers used for amplifying the V3-V4 hypervariable region of the bacterial 16S rRNA gene were 338F (5´-ACTCCTACGGGAGGCAGCAG-3′) and 806R (5´-GGACTACHVGGGTWTCTAAT-3′). The primers ITS1F (5´-CTTGGTCATTTAGAGGAAGTAA-3′) and ITS2R (5´-GCTGCGTTCTTCATCGATGC-3′) were used for amplification the internally transcribed spacer of fungal ITS region (Walters et al., 2018; Benitez et al., 2021). Amplicon sequencing was performed on an Illumina MiSeq PE300 platform (Illumina, San Diego, United States). The raw sequences were subjected to quality-control using fastq (Version 0.21.0) and merged using FLASH (Version 1.2.7) (Andrews, 2010). The UPARSE pipeline was then used to assign the sequences to operational taxonomy units (OTUs) based on 97% similarity level. Representative bacteria and fungi sequences were chosen and taxonomically assigned from genus to phylum by comparison with Silva database (Version 138.1) and UNITE databases (Version 9.0), respectively.

### 2.6 Statistical analysis

The metabolite and microbial data in rhizosphere soils were tested for normality and homogeneity of the variances. One-way ANOVA was used to analyse the significant differences in the agronomic traits of potatoes, the relative abundance of differential metabolites and major microbial phyla, alpha diversity, and soil physicochemical factors. Tucky’s test was then used to compare the means for each variable. Analyses of similarities based on Bray-Curtis distance matrices were used to evaluate differences in the community compositions of microbes and metabolites in rhizosphere soils among the study sites, and they were plotted using nonmetric multidimensional scaling (NMDS) ordination. Potentially differential soil microbial taxa among the study sites were screened by linear discriminant analysis.

The relationship between differential metabolites, soil edaphic factors, and soil microbial diversity in rhizosphere soils were determined by Pearson’s correlation analyses. Correlations between differential metabolites and soil microbial community compositions in rhizosphere soils were assessed using the Mantel test. Redundancy analysis (RDA) was used to investigate the relationship between soil edaphic factors and soil microbial community compositions. Variation partitioning analyses (VPA) were employed to determine the individual and common explanation rates of primary and secondary soil metabolites and soil factors in rhizosphere for the variation in microbial community compositions across different study sites. Furthermore, the co-variation between soil microbial community composition and secondary metabolites was examined using two-way orthogonal partial least squares regression (O2PLS) analysis, and the identity of the key drivers contributing to the co-variation was based on variable importance in projection (VIP ≥ 1). Data were statistically analyzed using SPSS 22.0 (IBM Corporation, Armonk, NY, United States), PAST software (version 4.03) and SIMCA (version 14.1). Plots were produced using R software (version 4.0.5) and Origin 2022 (OriginLab, Northampton, MA, United States).

## 3 Results

### 3.1 The agronomic characteristics of potatoes and rhizosphere soil physicochemical properties

**Figure 1:**
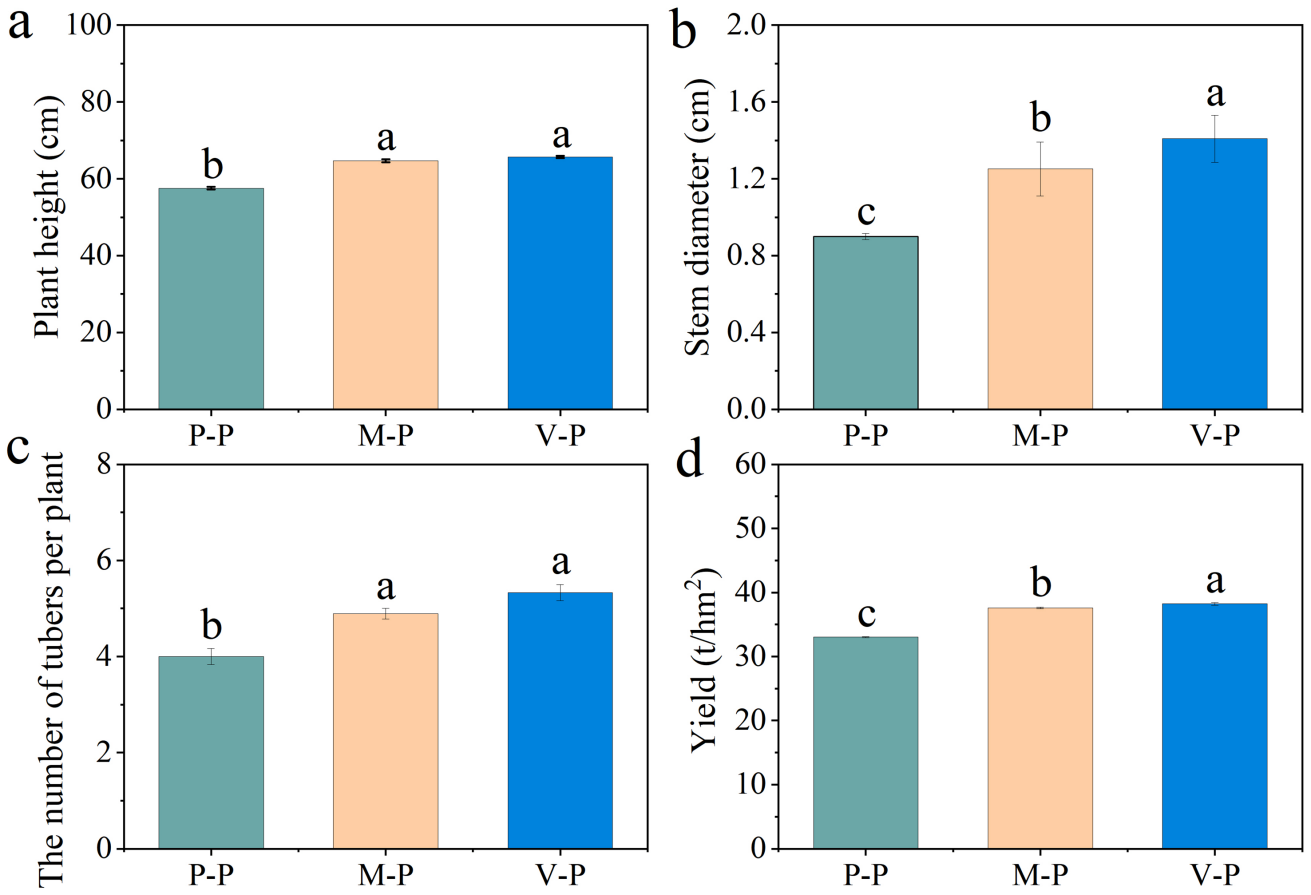
The agronomic traits of potatoes in different rotation systems. Error bars indicate the standard error. Different lowercase letters indicate significant differences between rotation patterns at *p* < 0.05. P-P, potato monoculture; M-P, maize-potato rotation; V-P, cowpea-potato rotation.

The height of the potatoes and the number of tubers per potato in the P-P rotation were significantly lower than that in the rotation systems. The stem diameter and yield of potatoes was lowest in the P-P rotation, but highest in the V-P rotation (Figure 1).

The pH of the rhizosphere soil and the amount of soil organic matter were highest in the V-P rotation. The content of soil total N in the M-P rotation was significantly higher than that in the P-P and V-P rotations, while soil total P content showed an opposite trend. Soil available P content was highest in the P-P rotation, but lowest in the V-P rotation (Table 1).

### 3.2 Metabolite profiles of potato rhizosphere soil

**Figure 2:**
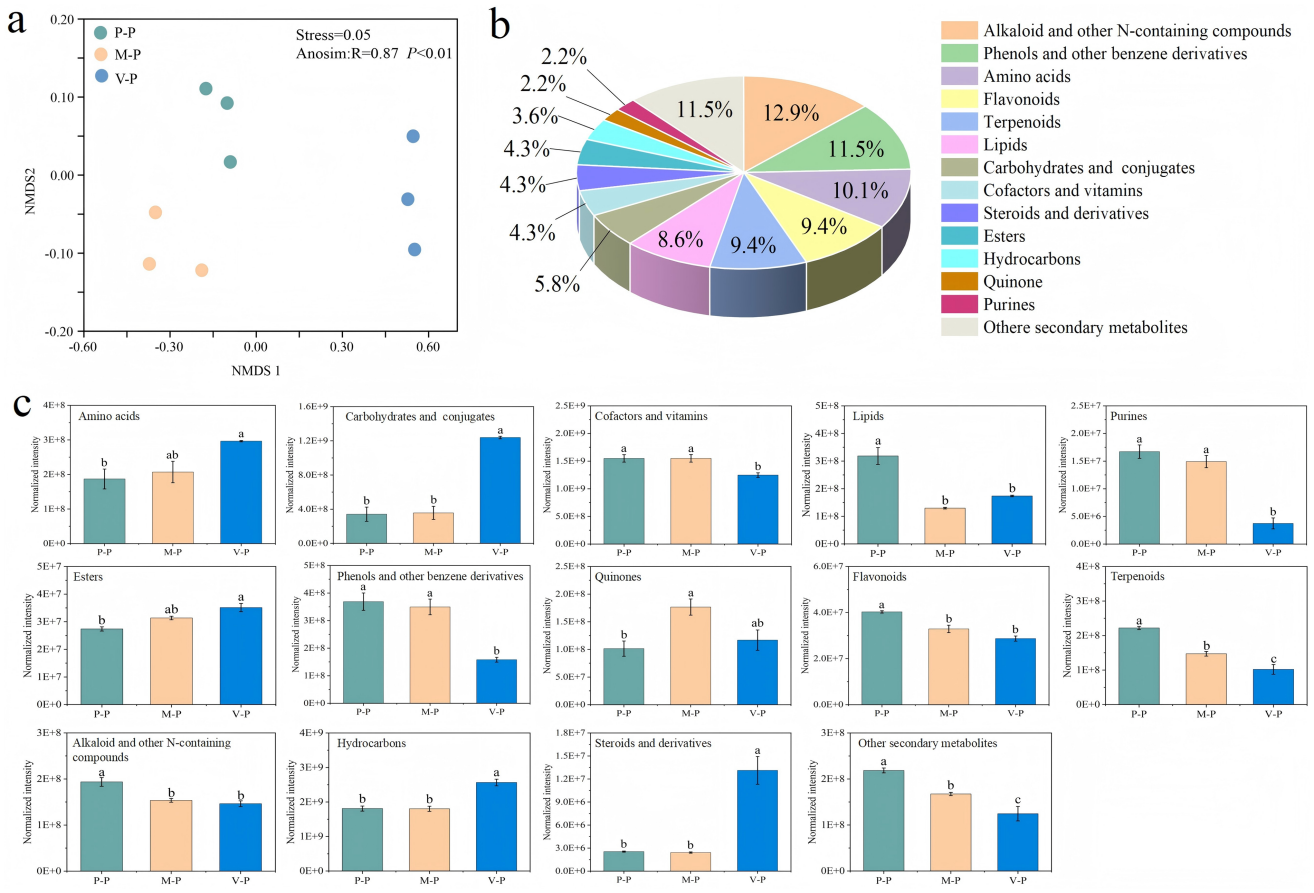
Nonmetric multidimensional scaling ordination for rhizosphere soil metabolites of potato in different rotation systems **(a)**. The categories of differential metabolites identified in rhizosphere soils of potato in the three rotation systems **(b)**. Normalized intensity of differential metabolites in rhizosphere soils of potato among different rotations **(c)**. Error bars indicate the standard error. Different lowercase letters indicate significant differences between rotation patterns at *p* < 0.05. For sample plot codes, refer to Figure 1.

A total of 978 metabolites including primary and secondary metabolites, and xenobiotics were identified in the potato rhizosphere soil, and their composition varied between continuous cropping and different rotation systems (ANOSIM: *R* = 0.87, *p* < 0.01) (Figure 2a). A total of 139 differentially expressed metabolites were detected in the potato rhizosphere soil across rotation systems. Compared to the P-P rotation, the levels of primary metabolites such as carbohydrates and conjugates, amino acids, and esters were increased by 3.63-, 1.56-, and 1.28-fold, respectively, in the V-P rotation. Cofactors and vitamins, lipids, and purines were present at the highest content in the P-P or M-P rotations (Figures 2b,c).

The main secondary metabolites related to defense mechanisms, such as alkaloid and other N-containing compounds (12.9%), phenols and other benzene derivatives (11.5%), flavonoids (9.4%), and terpenoids (9.4%) were decreased by 1.32-, 2.33-, 1.40-, and 2.17-fold, respectively, in the V-P rotation compared to the P-P rotation (Figures 2b,c). However, quinones were more abundant in the M-P rotation. The contents of hydrocarbons and steroids and derivatives significantly increased in V-P than in P-P and M-P rotations (Figure 2c).

### 3.3 Biodiversity of microorganisms in rhizosphere soil of potato

**Figure 3:**
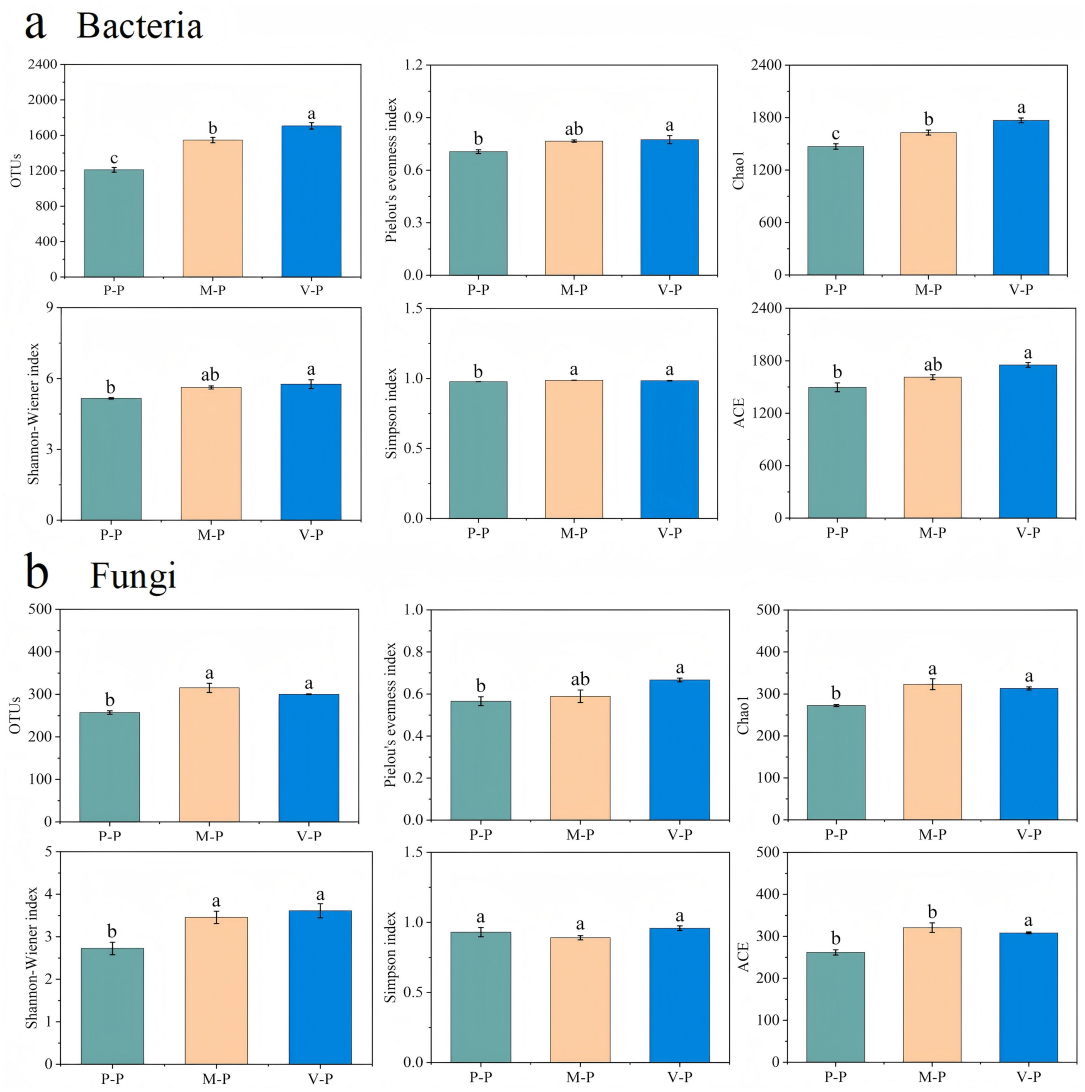
The OTUs and diversity index of bacteria **(a)** and fungi **(b)** in rhizosphere soils of potato in different rotations. Error bars indicate the standard error. Different lowercase letters indicate significant differences between rotation patterns at *p* < 0.05 For sample plot codes, refer to Figure 1.

The bacterial alpha-diversity was estimated at 1,210, 1,546, and 1,706 OTUs for potato rhizosphere soil samples in the P-P, M-P, and V-P rotations, respectively. The V-P rotation showed the highest Shannon and Chao1 indices, followed by the M-P rotation. The other diversity indices were lowest in the P-P rotation, but no significant differences were found between the M-P and V-P rotations (Figure 3a).

The number of fungal OTUs detected in potato rhizosphere soil was significantly higher in the M-P (315 OTUs) and V-P (300 OTUs) rotations than in P-P (257 OTUs) rotation. The highest values for the Shannon and Pielou’s indices were found in the V-P rotation, whereas the highest values for the Chao1 and ACE indices were found in the M-P rotation. However, all of these diversity indices showed no significant differences between V-P and M-P rotations (Figure 3b).

### 3.4 Community composition of microorganisms from rhizosphere soil of potato

The bacterial (*R* = 0.90, *p* < 0.01) and fungal (*R* = 0.95, *p* < 0.01) community compositions in potato rhizosphere soils were significantly different among rotation systems (Supplementary Figure S1). Of the top 10 most abundant bacteria phyla, Proteobacteria (45.55%), Bacteroidota (12.19%), Chloroflexi (6.08%), Gemmatimonadota (5.46%), and Myxococcota (1.06%) were the most abundant in the V-P rotation, and the least abundant in the P-P rotation. Patescibacteria (13.30%) and Firmicutes (4.58%) had a significantly higher relative abundance in the M-P rotation. However, Actinobacteria and Acidobacteria were more abundant in the P-P rotation (Supplementary Figure S2a; Table 2).

**Table 2:** ANOVA of the relative abundance (%) of the dominant bacterial and fungal phyla in rhizosphere soil of potato in different rotation systems.

Phyla	P-P	M-P	V-P
Bacteria			
Proteobacteria	34.22 ± 1.25c	39.55 ± 0.59b	45.55 ± 0.47a
Actinobacteriota	19.82 ± 1.42a	13.82 ± 0.04b	9.70 ± 0.10c
Bacteroidota	3.45 ± 0.16b	5.09 ± 0.104ab	12.19 ± 3.36a
Patescibacteria	8.71 ± 0.31b	13.30 ± 1.08a	10.04 ± 0.03b
Acidobacteria	17.64 ± 0.69a	8.13 ± 0.08b	3.99 ± 0.32c
Chloroflexi	2.32 ± 0.10b	5.65 ± 0.67ab	6.08 ± 1.28a
Gemmatimonadota	3.20 ± 0.60b	3.60 ± 0.49ab	5.46 ± 0.22a
Firmicutes	1.35 ± 0.17b	4.58 ± 0.11a	1.98 ± 0.38b
Cyanobacteria	0.87 ± 0.06a	0.84 ± 0.11a	1.40 ± 0.23a
Myxococcota	0.12 ± 0.01b	0.42 ± 0.09b	1.06 ± 0.22a
Fungi			
Basidiomycota	26.14 ± 0.66b	44.47 ± 2.08a	22.43 ± 2.54b
Ascomycota	22.90 ± 1.39a	25.23 ± 2.11a	23.77 ± 2.29a
Mortierellomycota	15.87 ± 2.42a	5.87 ± 2.42b	11.06 ± 1.54ab
Chytridiomycota	11.58 ± 0.15a	1.58 ± 0.15b	1.26 ± 0.24b
Rozellomycota	0.40 ± 0.19a	0.39 ± 0.18a	0.07 ± 0.02a
Aphelidiomycota	0.01 ± 0.01b	0.02 ± 0.00b	0.68 ± 0.45a
Monoblepharomycota	0.01 ± 0.01	0.01 ± 0.01	–
Basidiobolomycota	–	–	0.01 ± 0.00

Different lowercase letters indicate significant differences between rotation patterns at *p* < 0.05; For sample plot codes, see in Table 1.

Among the dominant fungal phyla, the relative abundance of Basidiomycota was significantly higher in the M-P rotation. Mortierellomycota and Chytridiomycota had a higher relative abundance in the P-P rotation. Aphelidiomycota were more abundant in the V-P rotation. No significant differences in the relative abundance of Ascomycota and Rozellomycota were observed between rotation systems. Additionally, Basidiobolomycota was detected in the V-P rotation, while Monoblepharomycota was detected in the M-P and P-P rotations (Supplementary Figure S2b; Table 2).

**Figure 4:**
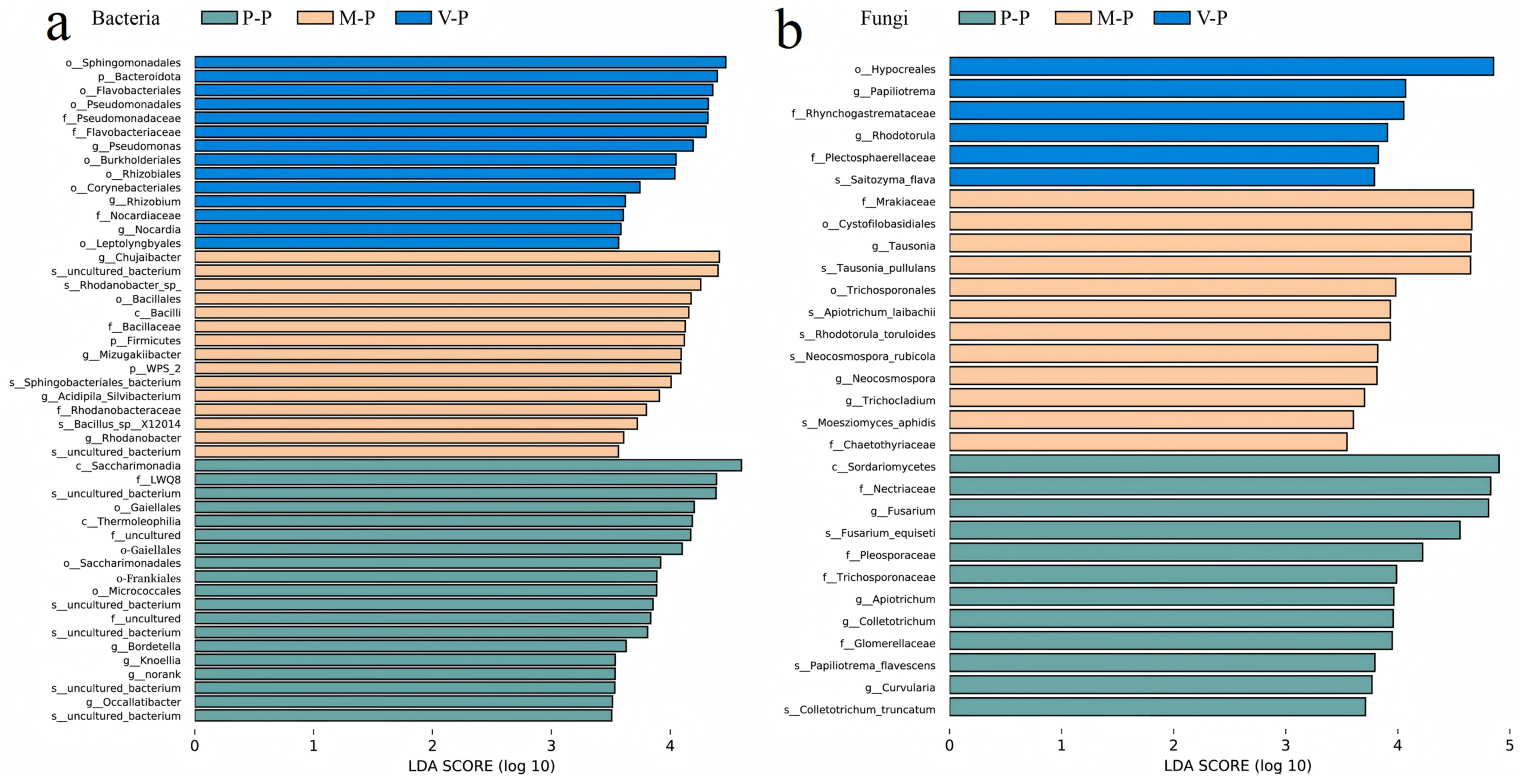
Linear discriminant analysis effect size identified the significantly different abundant bacterial **(a)** and fungal **(b)** taxa (biomarkers) at different taxonomic levels in potato rhizosphere soils between the rotations. For sample plot codes, refer to Figure 1.

Linear discriminant analysis identified 48 bacterial and 30 fungal communities with statistically significant abundance differences between rotation systems. Compared to P-P rotation, beneficial bacterial taxa were more abundant in rotation systems. Rhizosphere soils in the V-P rotation were enriched in P-solubilization bacteria family Pseudomonadaceae, the N-cycling bacteria genera *Rhizobium* and *Pseudomonas*, and the C-cycling bacteria order Burkholderiales, as well as pollutant-degrading bacteria order Sphingomonadales. The nutrient cycling-related bacteria genera *Chujaibacter*, *Rhodanobacter*, *Chujaibacter*, *Mizugakiibacter* and sepecies *Rhodanobacter* were more abundant in the M-P rotation. However, the P-P rotation contributed to an increased relative abundance of oligotrophic bacteria including the classes Thermoleophilia and Saccharimonadia, the order Frankiales, and genus *Knoellia*, as well as pathogenic genus *Bordetella* (Figure 4a).

The fungal taxa with biocontrol potential including the order Hypocreales, genus *Rhodotorula*, and symbiotic fungi species *Saitozyma_flava* had higher relative abundance in the V-P rotation. However, the P-P rotation increased the relative abundance of pathogenic fungal taxa, including the family Plectosphaerellaceae, Pleosporaceae and Trichosporonaceae, genera *Colletotrichum* and Curvularia, species *Fusarium equiseti* and *Colletotrichum truncatum*. The relative abundance of genus *Tausonia*, species *Rhodotorula toruloides* and *Apiotrichum laibachii* were enriched in the M-P rotation (Figure 4b).

### 3.5 Associations of rhizosphere metabolites, soil properties with the microbial diversity and community compositions

**Figure 5:**
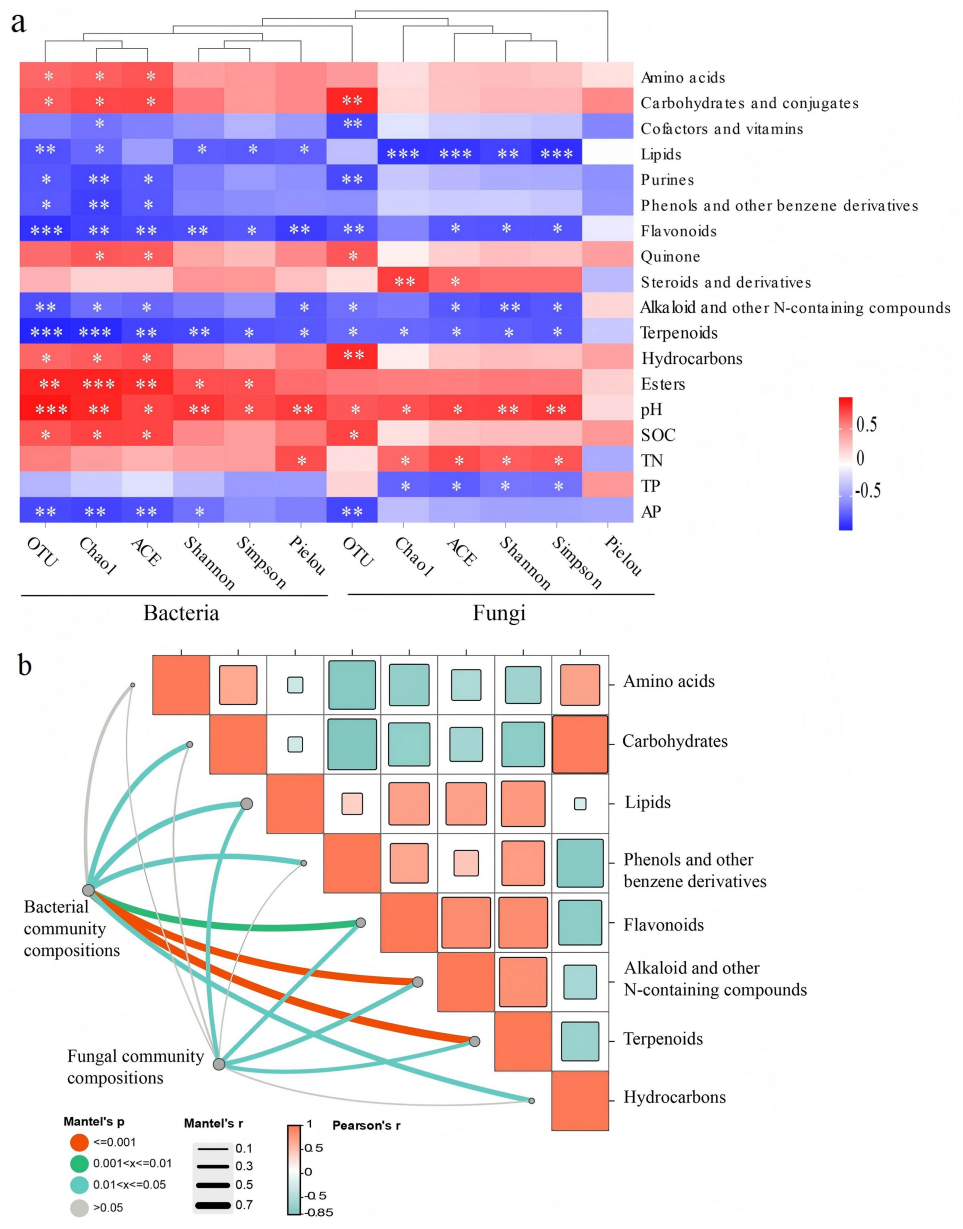
Pearson’s correlation analysis of differential metabolites and soil physicochemical characteristics (pH: soil pH, SOM: soil organic matter, TN: soil total N, TP: soil total phosphorus; AP: soil available phosphorus) with the diversity of soil microbiome in rhizosphere soil of potato **(a)**. Mantel test evaluating the relationships between main differential rhizosphere metabolites and microbial community composition **(b)**. Significance level: ^*^p < 0.05, ^**^p < 0.01, ^***^p < 0.001.

Microbial diversity was positively correlated with the content of amino acids and cofactors and vitamins, but negatively correlated with lipids and purines content. Secondary metabolites including flavonoids, terpenoids, alkaloid and other N-containing compounds, and phenols and other benzene derivatives predominantly negatively correlated with diversity indices. Rhizosphere soil pH, soil organic matter, and total N were positively related with microbial diversity. Soil total P and available P was negatively related with bacterial and fungal diversity, respectively (Figure 5a).

**Figure 6:**
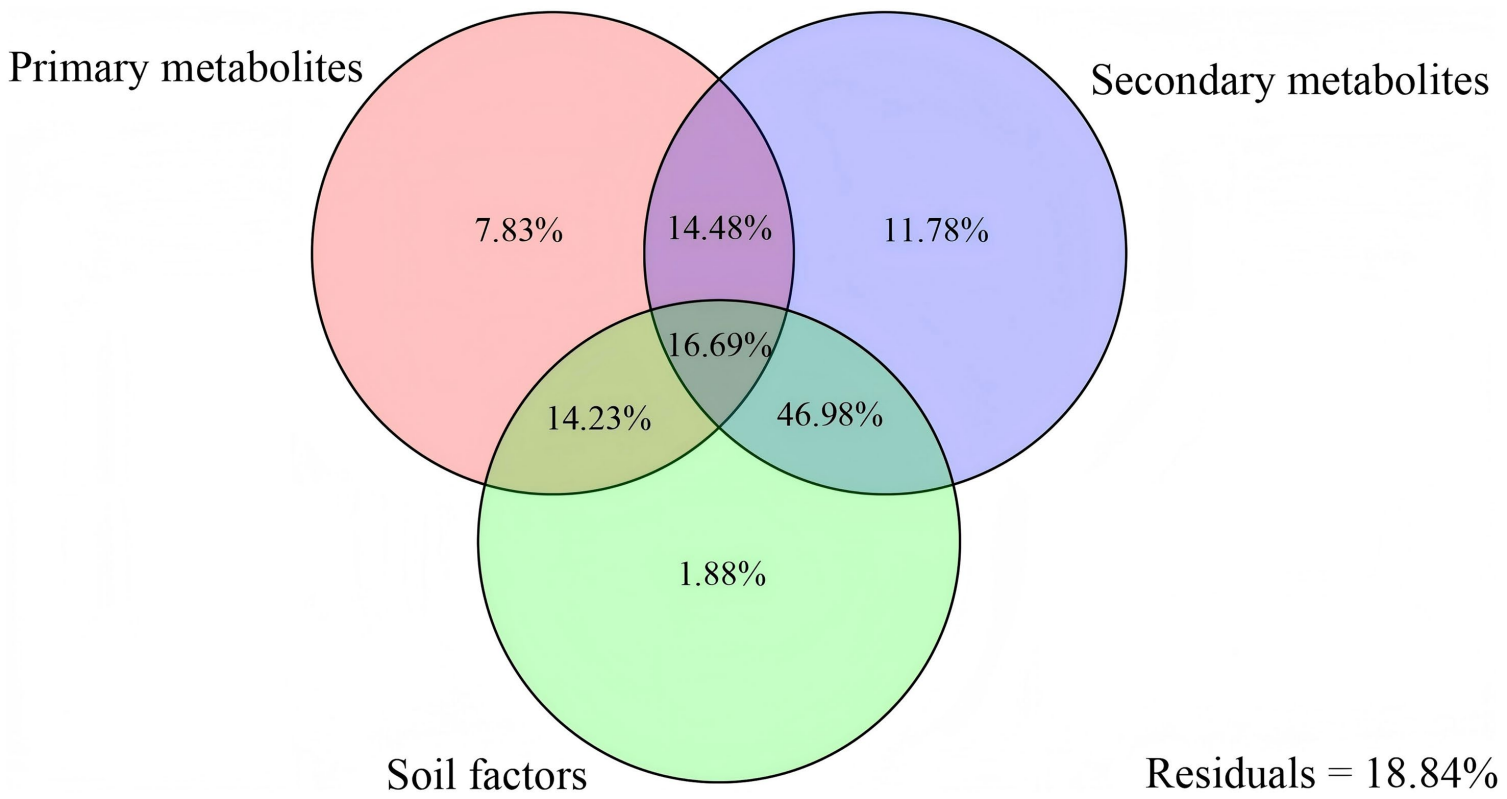
Variation partitioning analysis of the effects of primary metabolites, secondary metabolites, soil factors of potato rhizosphere soils on the soil microbial community composition dissimilarity.

**Figure 7:**
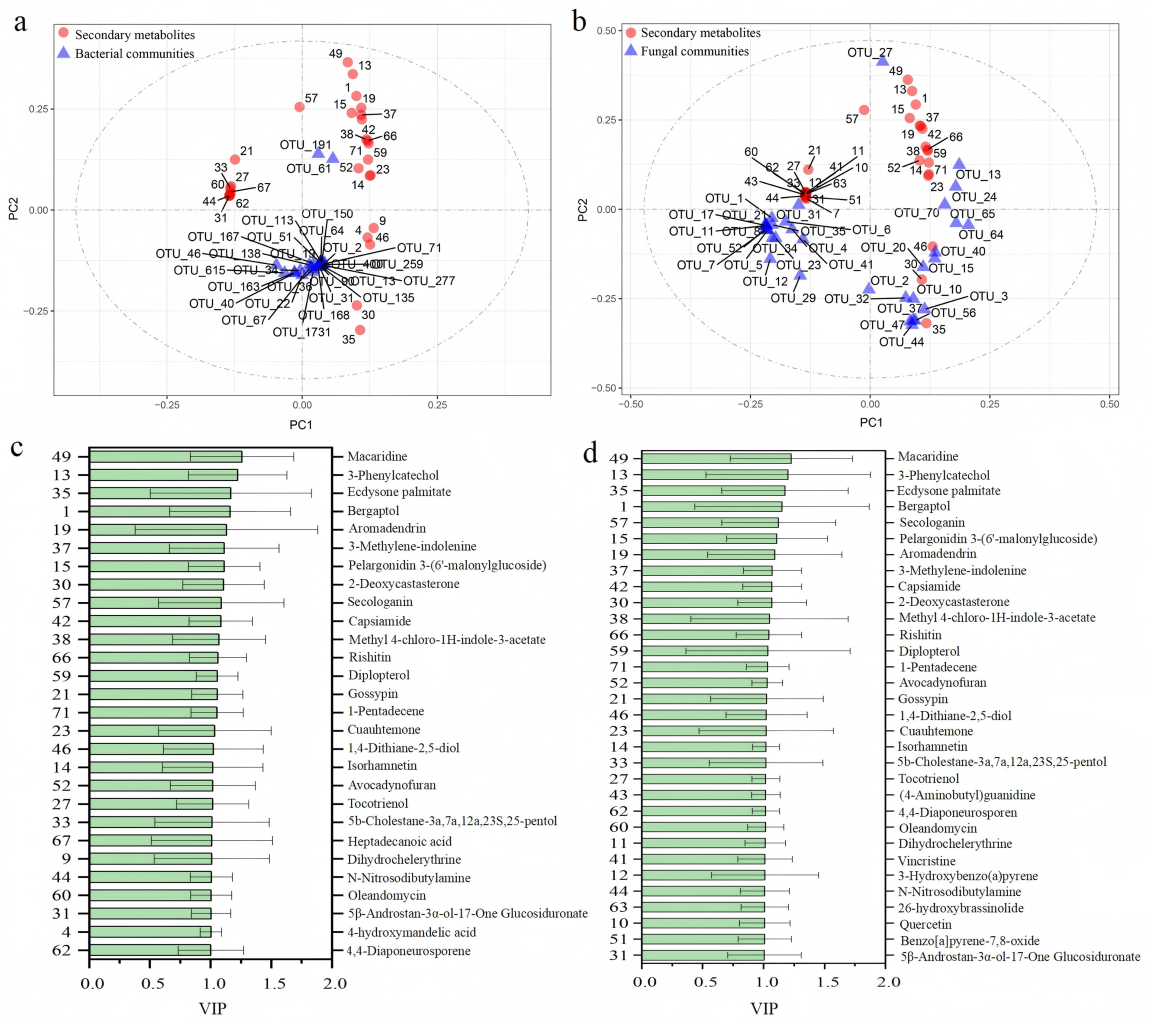
Two-way orthogonal partial least squares (O2PLS) score plot showing the association between characteristic differential secondary metabolites and soil bacteria **(a)** and fungi **(b)**. The VIP of each secondary metabolite was arranged from high to low **(c,d)**. Only metabolites with VIP values greater than 1 were presented.

The Mantel test results showed that the composition of the bacterial community in rhizosphere soil was significantly related to all rhizosphere metabolites except for amino acids. Fungal community compositions were significantly related to lipids and flavonoids, alkaloids and other N-containing compounds, and terpenoids. Flavonoids, alkaloids and other N-containing compounds, and terpenoids exhibited a strong influence on microbial community compositions than other metabolites (Figure 5b). Further variation partitioning analysis founded that secondary metabolites dominated the microbial community composition variation, accounting for 89.93% of the variations (Figure 6). The O2PLS analysis with Q^2^ values of 0.50 and o.51 identified 28 and 33 secondary metabolites (VIP > 1) (Figures 7a,b). These metabolites predominantly belonged to the major classes of alkaloids, terpenoids, and flavonoids. The most key drivers (VIP > 1.1) of variance in microbial structure were macaridine, 3-phenylcatechol, Ecdysone palmitate, bergaptol, pelargonidin, and aromadendrin (Figures 7c,d).

## 4 Discussion

### 4.1 Crop rotation modulates rhizosphere soil metabolites compositions of potato

Our findings supported the hypothesis that crop rotation triggers changes in the abundance and composition of metabolites in the rhizosphere soils of potato. Main primary metabolites identified in our study such as amino acids and carbohydrates were more abundant in the rotation system, especially in cowpea and potato rotation. Amino acids and carbohydrates metabolism is associated with the C and N cycles and predominantly reflects soil nutrient accumulation and the microbial activity (Roberts et al., 2009; Geisseler et al., 2010; Zhang D. J. et al., 2022; Zhang J. X. et al., 2022). Our result suggested that potato rotation may promote C, N, and other nutrient cycling in rhizosphere soils. However, lipids had a higher content in rhizosphere soils of continuous potato crops. Lipids play an essential role in forming the cell membranes and storing energy, and lipid metabolisms regulation is one of the mechanisms by which biota adapt to adverse conditions (Lu et al., 2019; Hall et al., 2020; Wang Y. et al., 2023; Wang Y. Z. et al., 2023). Continuous potato cropping could result in lower soil pH levels, nutrient depletion, and the accumulation of toxic substances, which can further induce disorder of microbial structure, favoring pathogen taxa (Larkin et al., 2021; Qin et al., 2022; Ma et al., 2023; Wang Y. et al., 2023; Wang Y. Z. et al., 2023). These adverse conditions may influence lipids metabolism of potatoes and associated microorganisms, which could explain their higher abundance under successive potato crops.

Major secondary metabolites including phenols, flavonoids, terpenoids, and alkaloids and their derivatives, exhibited significantly lower abundance in the cowpea and potato rotation, but higher abundance in continuous cropping. Most of the identified secondary metabolites, such as quercitrin, genistein, macaridine, and dihydrochelerythrine, act as allelochemicals and may participate in chemical defense mechanisms of crops (Cao et al., 2022; Goławska et al., 2024; Pan et al., 2024). Rishitin, a sesquiterpene phytoalexin, has been well documented as a key defensive metabolite in the Solanaceae family such as tomatoes and potatoes, against plant pathogens particularly *Phytophthora infestans* which cause potato late blight (Gardner et al., 1994; Bulasag et al., 2024). In our study, rishitin content in rhizosphere soils was highest in continuous potato cropping, but lowest in the cowpea and potato rotation. These results indicate that potato rotation may alleviate the intensified pathogen pressure of successive crops, emphasizing the direct influence of rotation management on the diseases mitigation. The shift from defensive to primary metabolites may reflect the fact that potatoes reallocate more resources from defense to growth in rotation systems. Previous studies have demonstrated the positive effects of potato rotation with leguminous crops mediated by various mechanisms (Yigezu et al., 2019; Wang Y. et al., 2023; Wang Y. Z. et al., 2023; Xu et al., 2025). For example, in faba-potato rotation systems, soil quality and enzyme activity were increased, and beneficial microbial colonization against disease were promoted (Qin et al., 2022; Wang Y. et al., 2023; Wang Y. Z. et al., 2023). Consistent with these study results, we also found stem diameter, the number of tubers per potato, and yield of potatoes were highest in cowpea and potato rotation. Our results of significant decrease in defense secondary metabolites may also provide support for beneficial effects in potato with leguminous crop rotation systems.

### 4.2 Crop rotation alters rhizosphere soil microbial communities

Our study found that rotation cropping enhances bacterial and fungal diversity and induces changes in microbial community composition in potato rhizosphere soils. The dominant bacterial phyla Proteobacteria and Bacteroidota have been identified as copiotrophic taxa and their relative abundance were found to be positively correlated with soil pH (Qu et al., 2020). The higher levels of soil organic carbon and pH in the potato rhizosphere soil may explain their higher relative abundance in rotation systems compared to continuous cropping (Supplementary Figure S3). Some biocontrol, nitrogen-fixing, denitrification, methane-metabolism, and pollutant-degradation bacteria such as genus *Pseudomonas*, family Rhizobiaceae, orders Sphingomonadales and Burkholderiales, and some other taxa involved in nutrient cycling such as genera *Chujaibacter*, *Rhodanobacter*, *Mizugakiibacter*, and species *Rhodanobacter* were more abundant in rotation systems. These results demonstrate that crop rotation significantly enriches beneficial bacterial taxa in the rhizosphere soils.

Some fungal taxa that could cause disease were also identified in potato rhizosphere soils and changes between rotation patterns. The dominance of pathogen-related taxa, e.g., the genus *Fusarium*, is associated with the devastating plant diseases such as Fusarium wilt, root rot, and head blight (Davis et al., 2009). The genera *Colletotrichum* and *Curvularia* can cause anthracnose and leaf spot diseases in many crops such as soybean, maize, and wheat (Wonglom et al., 2019). In our study, potato rotation significantly reduced the relative abundance of these pathogens. Together with the changes in bacterial communities, these results may suggest a shift from fungal to bacterial-dominated communities in rhizosphere soils following potato rotation. Previous studies on crop rotation have extensively reported that *Pseudomonas* spp. in rhizosphere soils can suppress *Fusarium oxysporum* populations, mainly by mechanisms such as the production of siderophore (pyoverdine), phenazine production, the upregulation of defense genes, as well as by nutrient and spatial competition (de Boer et al., 2003; Caulier et al., 2018; Xu et al., 2025). Therefore, the observed lower abundance of pathogen-related taxa in potato rotation systems may be closely associated with microbial antagonism. Selecting an appropriate crop rotation with potato has the potential to control soil borne diseases occurring in successive cropping systems.

### 4.3 Rhizosphere soil metabolites are closely related to soil microbiomes

Our findings revealed significant relationships between the diversity and community composition of rhizosphere microbes and potato rhizosphere metabolites composition. Amino acids and carbohydrates exhibited positive correlations with the diversity and community composition of bacteria, which may be consistent with their roles in providing readily nitrogen and available carbon to support the chemotaxis of specific beneficial bacteria (Geisseler et al., 2010; Zhang D. J. et al., 2022; Zhang J. X. et al., 2022; Lv et al., 2025). Studies have found that fatty acids and their derivatives from lipid metabolism such as palmitic acid, stearic acid, and oleic acid could exhibit antimicrobial properties (Obukhova and Murzina, 2024). Consistent with these previous studies, we observed negative association between lipids and microbial diversity and community composition in potato rhizosphere soils.

The results revealed that secondary metabolites play a dominant role in regulating microbial communities in potato rhizosphere soils. Among them, flavonoids, alkaloids, and terpenoids exhibited a stronger association with variations in bacterial and fungal community compositions. These compounds are known to possess antimicrobial or signaling properties, which could directly reshape microbial communities (Ankala et al., 2013; Kong et al., 2019; Cao et al., 2022). For example, terpenoids can inhibit the respiration or enzyme activity involved in nutrient acquisition by microorganisms such as beneficial genus *Bacillus* (Kong et al., 2007). Alkaloids would directly penetrate the cell membranes of microorganisms, destroying their structure and function (Cushnie et al., 2014). Flavonoids could damage microbial cells by triggering oxidative stress responses (Perz et al., 2024). In our study, identified secondary metabolites such as rishitin, diplopterol, oleandomycin, 3-Methylene-indolenine, and quercitrin all have allelopathic potential on microorganisms, thereby influencing their relative abundance (Cao et al., 2022; Bulasag et al., 2024; Pan et al., 2024). Rhizosphere secondary metabolites also act as signaling molecules, which exert diverse effects on soil microbiomes, including promotion, inhibition, and eviction (Bais et al., 2006; Kong et al., 2019; Sikder and Vestergård, 2019; Yue et al., 2023). In our study, rotating potato cultivation had a positive effect on the growth and yield of potatoes. This may be due to changes in the rhizosphere processes, reducing the amount of defensive secondary metabolites and increasing the number of beneficial microorganisms. Our results revealed the regulation of rhizosphere metabolites on microbial communities. This study provides a unique insight into the mechanisms underlying the benefits of crop rotation by modulating the interactions between rhizosphere metabolites and microbiomes.

There are several limitations in the present study. At first, single sampling time point was unable to capture the dynamic changes in rhizosphere metabolites and soi microbial communities, restricting the elucidation of potential mechanisms driving changes and a comprehensive understanding of rotation effects. Future work incorporating multi-growth stage sampling is essential to fully reveal crop rotation effects. Secondly, the absence of pre-planting soil physicochemical properties limits the results. Future studies should include baseline soil property assessments to better quantify the effects of crop rotation. Finally, the relatively small sample size may constrain the statistical power. Expanding the sample size in future work are needed.

## 5 Conclusion

Our results showed that crop rotation had a positive effect on the growth and yield of potatoes. There were significant differences in rhizosphere soil metabolites between potato-potato, maize-potato, and cowpea-potato crop rotations. Compared with continuous potato cultivation, the rhizosphere soil metabolites of potato in rotation systems had fewer defensive secondary metabolites, but more abundant primary metabolites. In potato rotation systems, the diversity of rhizosphere soil bacteria and fungi increased and microbial community compositions significantly changed. More beneficial microbial taxa involved in biocontrol, nitrogen fixation, C degradation, methane metabolism, denitrification, and pollutant degradation bacteria were enriched in rotation systems, while the abundance of dominant pathogenic microbial communities were decreased, especially in the cowpea-potato rotation. Rhizosphere soil metabolites were significantly related to the diversity and composition of soil microbiome. Specific secondary metabolites with defensive functions including flavonoids, alkaloids, and terpenoids dominated the variation of the soil microbial community composition. These findings highlight that the changes in rotation-driven rhizosphere metabolites probably shape soil microbiomes. This study indicated the potential interaction between rhizosphere metabolites and microbiomes, providing more comprehensive theoretical support for the benefits of crop rotation.

## Data Availability

The datasets presented in this study can be found in online repositories. The names of the repository/repositories and accession number(s) can be found here: https://ngdc.cncb.ac.cn/bioproject/browse/PRJCA053159.

## Footnotes

1
http://www.hmdb.ca

2
http://www.massbank.jp/

3
https://www.genome.jp/kegg/

4
http://www.lipidmaps.org

5
https://www.mzcloud.org
