# Supplementary material for: Crop rotation-driven changes in secondary metabolites of potato rhizosphere soil exert stronger regulation on soil microbial community
Source: Front Microbiol. 2026 Mar 5;17:1768797. doi: 10.3389/fmicb.2026.1768797 (PMC12999584; doi:10.3389/fmicb.2026.1768797)
Supplement: Supplementary file 1 [file Data_Sheet_1.docx]

*Supplementary Material*

**Crop rotation-driven changes in secondary metabolites of potato rhizosphere soil exert stronger regulation on soil microbial community**

Jinjin Li ^1, †,^ *, Qingcheng Li ^2, †^, Mantang Wang ^1^, Shuqing Xu ^1^, Danju Zhang ^3^

^1^ College of Tourism, Resources and Environment, Zaozhuang University, Zaozhuang, China

^2^ Triticeae Research Institute, Sichuan Agricultural University, Chengdu, Sichuan, China

^3^ College of Forestry, Sichuan Agricultural University, Chengdu, Sichuan, China

^*^ Correspondence: jjl_0907@126.com

**Supplementary Table 1.** The total fertilization amounts and fertilizer types of three rotation regimes.

| Crop | Potato season (kg hm-2) |  |  | Crop rotation season (kg hm-2) | | |
| --- | --- | --- | --- | --- | --- | --- |
|  | N | P2O5 | K2O | N | P2O5 | K2O |
| Potato | 200 | 120 | 220 | / | / | / |
| Maize | 200 | 120 | 220 | 230 | 90 | 120 |
| Cowpea | 200 | 120 | 220 | 105 | 135 | 180 |


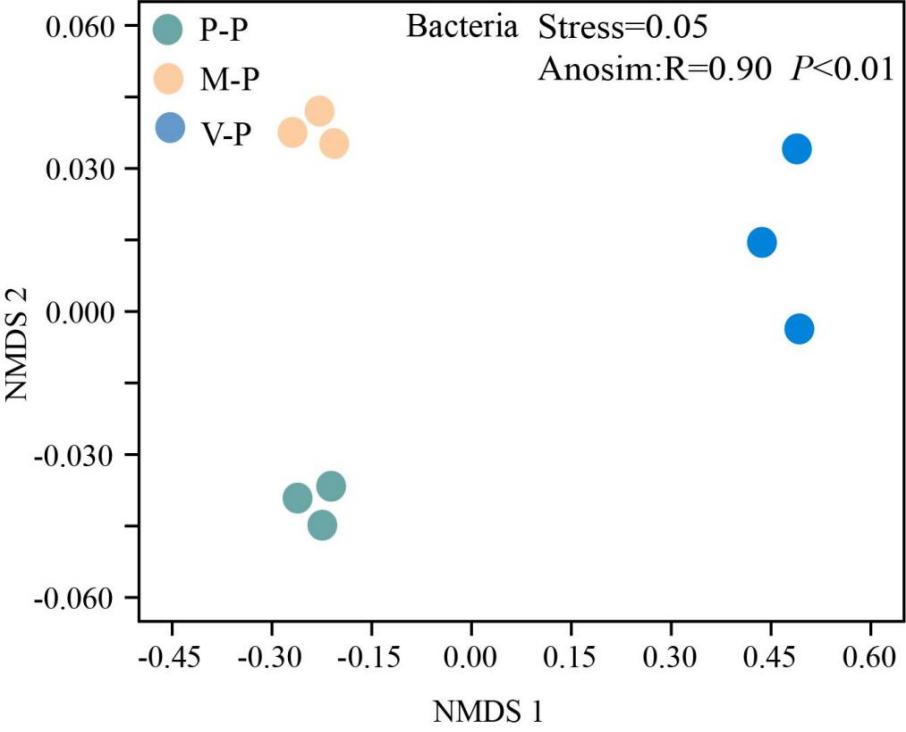

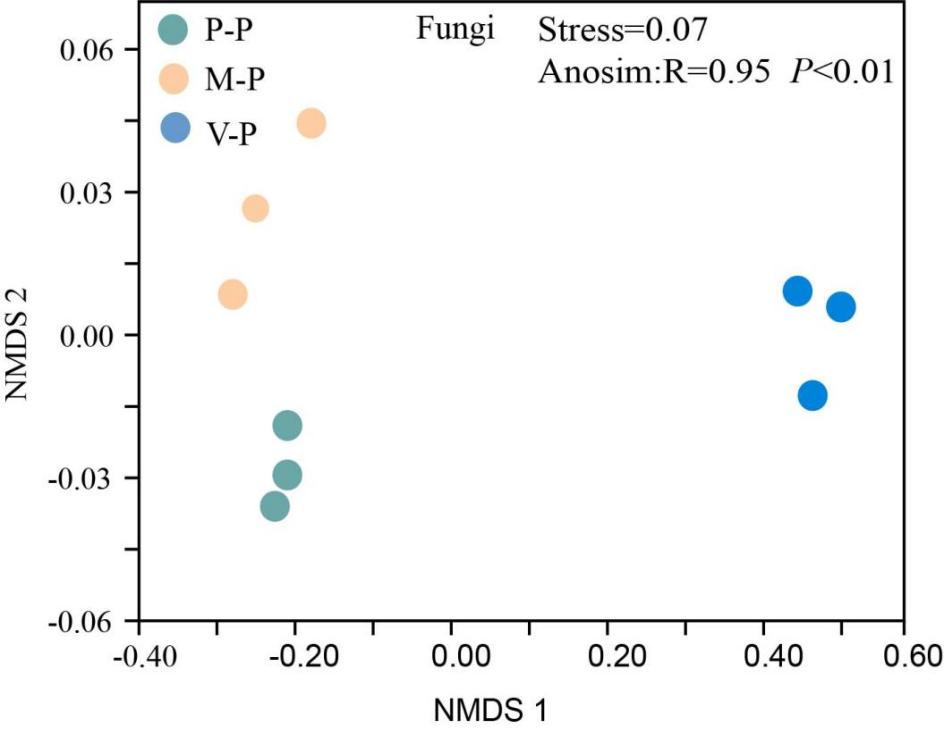


a

b

**Supplementary Figure 1.** Nonmetric multidimensional scaling ordination for the community composition of bacteria (a) and fungi (b) in rhizosphere soils of potato in different rotations. P-P, potato monoculture; M-P, maize-potato rotation; V-P, cowpea-potato rotation.


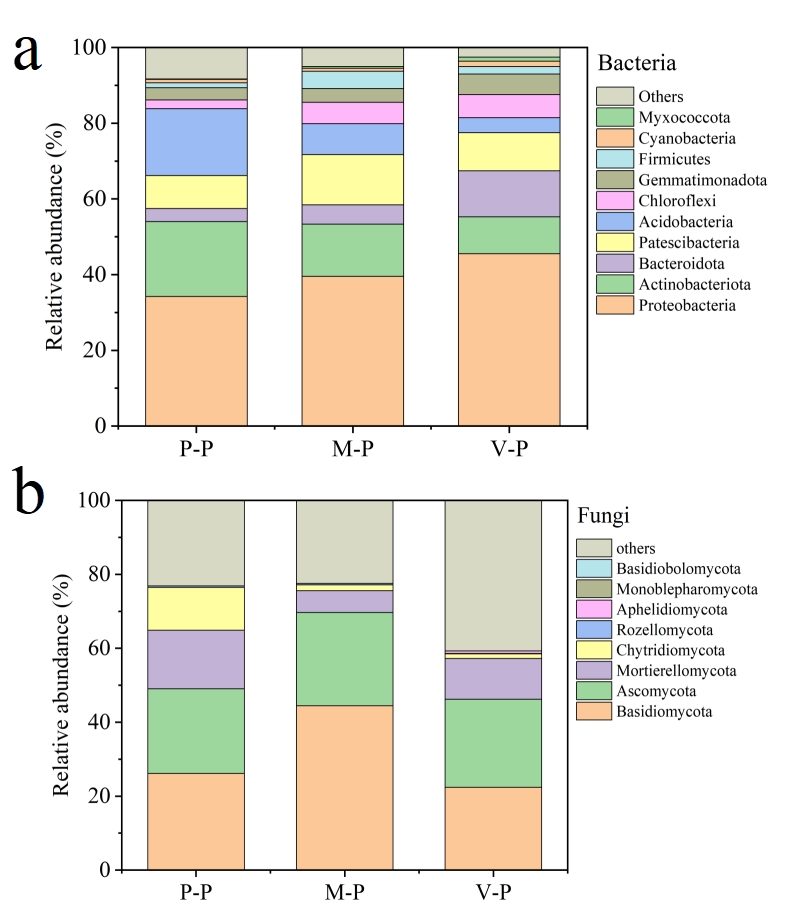


**Supplementary Figure 2.** The relative abundance of bacteria (a) and fungi (b) at the phylum level from the potato rhizosphere soil in different rotations. P-P, potato monoculture; M-P, maize-potato rotation; V-P, cowpea-potato rotation.


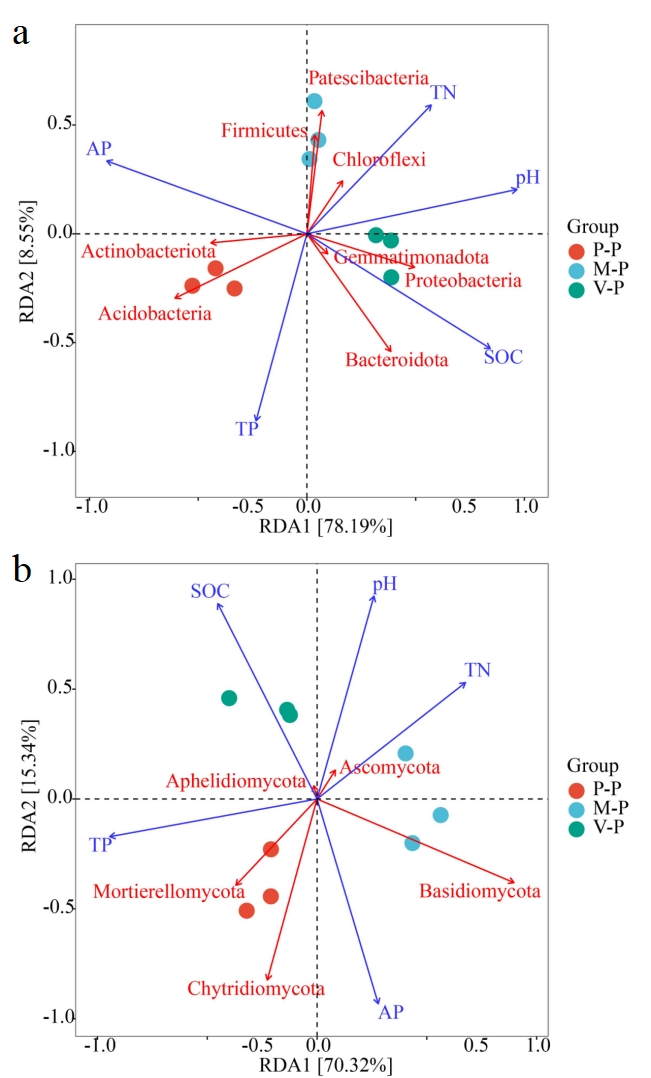


**Supplementary Figure 3.** Redundancy analysis (RDA) investigating the relationship between soil physicochemical properties and relative abundance of bacteria (a) and fungi (b) at the phylum level in rhizosphere soil of potato. P-P, potato monoculture; M-P, maize-potato rotation; V-P, cowpea-potato rotation.
